# 

**Published:** 1904-10

**Authors:** 


VOL. XX. OCTOBER, 1904. No. 4.

LIBRARY
INDEXED
S.G.O.

### TEXAS Medical Journal.

SUBSCRIPTION, $1.00 A YEAR, IN ADVANCE.

SINGLE COPIES, 10 CENTS.

LIBRARY
SURGEON GENERAL'S OFFICE
OCT. 3 - 1904

PUBLISHED AT AUSTIN, TEXAS, BY
F. E. DANIEL, M. D.,
PROPRIETOR.

PRINTED BY THE VON BOECKMANN-JONES CO.

Entered at the Postoffice at Austin, Texas, as Second-class Mail Matter.

